# Supplementary material for: RA-XII Suppresses the Development and Growth of Liver Cancer by Inhibition of Lipogenesis via SCAP-dependent SREBP Supression
Source: Molecules. 2019 May 12;24(9):1829. doi: 10.3390/molecules24091829 (PMC6539016; doi:10.3390/molecules24091829)
Supplement: Supplementary file 1 [file molecules-24-01829-s001.pdf]

*Supplementary information*

# RA-XII Suppresses the Development and Growth of Liver Cancer by Inhibition of Lipogenesis via SCAP-dependent SREBP Supression

**Di Guo [1,†], Yurong Wang [1,†], Jing Wang [1], Lihua Song [1], Zhe Wang [1,2], Bingyu Mao [3] and Ninghua Tan [1,2,*]**

1 State Key Laboratory of Natural Medicines, Department of TCMs Pharmaceuticals, School of Traditional Chinese Pharmacy, China Pharmaceutical University, 211198 Nanjing, China; guodi33@163.com (D.G.); yurong1987213@163.com (Y.W.); 18851107621@163.com (J.W.); songlihua4835@163.com (L.S.); wangzhe153807105@163.com (Z.W.)

2 State Key Laboratory of Phytochemistry and Plant Resources in West China, Kunming Institute of Botany, Chinese Academy of Sciences, 650201 Kunming, China

3 State Key Laboratory of Genetic Resources and Evolution, Kunming Institute of Zoology, Chinese Academy of Sciences, 650223 Kunming, China; mao@mail.kiz.ac.cn

* Correspondence: nhtan@cpu.edu.cn; Tel.: +86-25-8618-5772

† These authors contributed equally to this work.

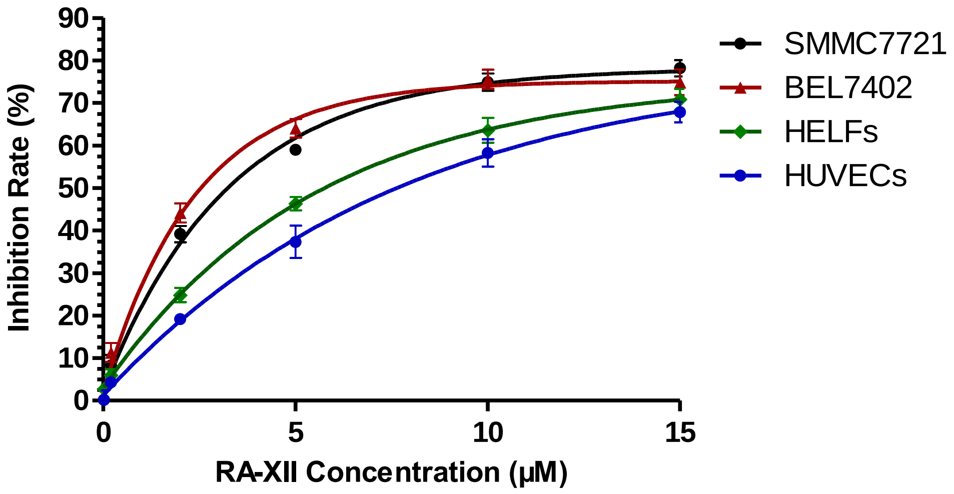


**Figure S1.** SRB measurement of cell vitality. Cells were treated with different concentrations of RA-XII for 48 h. Results are means ± SEM of three independent experiments.

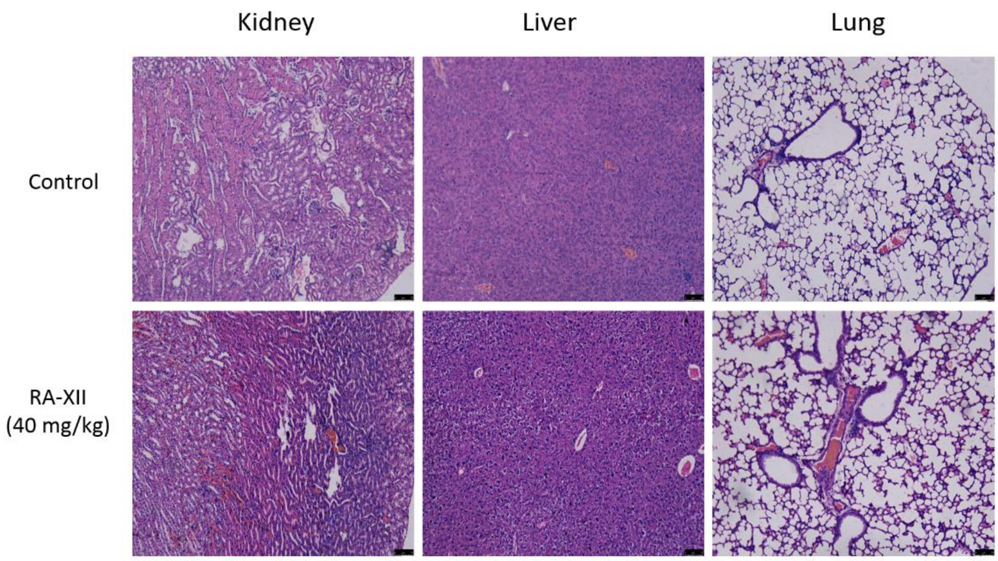


**Figure S2.** Hematoxylin and eosin staining of kidney, liver and lung sections of the xenograft mouse model (Scale bar: 75 μm).

Empower 3 SOFTWARE

# Overlay Report

## SAMPLE INFORMATION

| | | | |
|---|---|---|---|
| Sample Name: | RA-XII KM-2 | Acquired By: | System |
| Sample Type: | Standard | Sample Set Name | 2 |
| Vial: | 2:A,2 | Acq. Method Set: | 30min_yanchun |
| Injection #: | 1 | Processing Method | Default |
| Injection Volume: | 5.00 ul | Channel Name: | 2998 Ch1 210nm@4.8nm |
| Run Time: | 30.0 Minutes | Proc. Chnl. Descr.: | 2998 Ch1 210nm@4.8nm |
| Date Acquired: | 9/22/2018 5:06:05 PM CST | | |
| Date Processed: | 4/26/2019 4:19:12 PM CST | | |

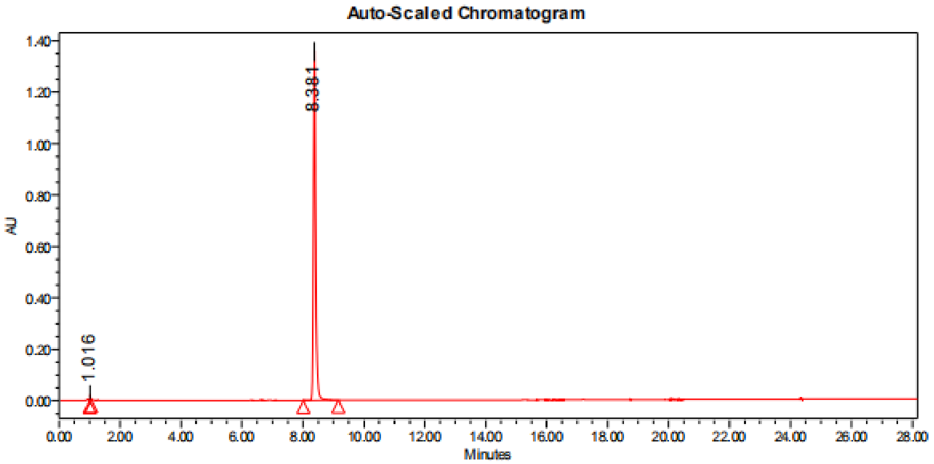


### Processed Channel: 2998 Ch1 210nm@4.8nm

| | Processed Channel | Retention Time (min) | Area | % Area | Height |
|---|---|---|---|---|---|
| 1 | 2998 Ch1 210nm@4.8nm | 1.016 | 23161 | 0.34 | 21849 |
| 2 | 2998 Ch1 210nm@4.8nm | 8.381 | 6776458 | 99.66 | 1360928 |



**Figure S3.** HPLC-DAD chromatogram of RA-XII, which indicates that RA-XII used in this study is pure.
